# Outcome comparison of meniscal allograft transplantation (MAT) and meniscal scaffold implantation (MSI): a systematic review

**DOI:** 10.1097/JS9.0000000000001587

**Published:** 2024-05-13

**Authors:** Jize Dong, Moran Huang, Jinrong Lin, Yaying Sun, Xingyu Zhang, Jiwu Chen

**Affiliations:** a Department of Sports Medicine, Shanghai General Hospital Affiliated to Shanghai Jiao Tong University, Hongkou District; b Department of Sports Medicine, Huashan Hospital, Fudan University, Shanghai, China

**Keywords:** allograft transplantation, meniscus tear, scaffold implantation

## Abstract

**Background:**

Although numerous studies have reported successful clinical outcomes of meniscal allograft transplantation (MAT) or meniscal scaffold implantation (MSI), the difference between the outcome of MAT and MSI remains unclear.

**Purpose:**

To compare the overall outcomes and survival rates of MAT and MSI, aiming to provide comprehensive evidence for determining the optimal treatment strategy for meniscal defects.

**Methods:**

A systematic review was performed via a comprehensive search of PubMed, Embase, and the Cochrane Library. Studies of MAT or MSI were included according to the inclusion and exclusion criteria. The Lysholm score was chosen as the primary outcome measure, while secondary outcomes encompassed patient-reported outcome measures (PROMs), return to sports (RTS) rates, survival rates, and complication rates. The outcomes were stratified into two groups: MAT group and MSI group, followed by statistical comparison (*P*<0.05). The quality of the included studies was assessed by the Cochrane Risk of Bias 2 (RoB2) assessment tool for randomized controlled trials (RCTs) and the Coleman Methodology Score (CMS) for non-randomized controlled trials.

**Results:**

A total of 3932 patients (2859 MAT, 1073 MSI) in 83 studies (51 MAT, 32 MSI) had the overall significant improvement in all clinical scores. The group MSI had a higher Lysholm score of both preoperative (*P*=0.002) and postoperative (*P*<0.001) than group MAT; however, the mean improvements were similar between the two groups (*P*=0.105). Additionally, MSI had higher improvements of IKDC (*P*<0.001), KOOS symptom (*P*=0.010), KOOS pain (*P*=0.036), and KOOS ADL (*P*=0.004) than MAT. Interestingly, MAT had higher preoperative (*P*=0.018) and less postoperative VAS pain (*P*=0.006), which was more improved in MAT (*P*<0.001). Compared with MAT, MSI had a higher 10-year survival rate (*P*=0.034), a similar mid-term survival rate MAT (*P*=0.964), and a lower complication rate (*P*<0.001).

**Conclusion:**

Both MAT and MSI could have good clinical outcomes after surgery with a similar improvement in Lysholm score. MSI had a higher 10-year survival rate and fewer complications than MAT.

**Level of evidence:**

Level IV, systematic review.

## Introduction

Highlights

We compared the overall outcomes and survival rates of meniscal allograft transplantation (MAT) and meniscal scaffold implantation (MSI).

As much as 83 studies with 3705 patients in total were included.

Both MAT and MSI could have good clinical outcomes after surgery with the similar improvement in Lysholm score.

MSI had a higher 10-year survival rate and fewer complications than MAT.

Considering the important role in knee joint function^1^, meniscal preservation following injury should be prioritized^2^. However, severe damage can result in significant structural and functional deficits^3,4^, necessitating meniscus transplantation with allografts for young active patients to restore load-bearing capacity and chondroprotective effects^5,6^.

The partial meniscus defect, even with intact peripheral rim, anterior and posterior horn, has been reported to have the risk of long-term cartilage destruction^7^. Therefore, partial meniscus transplantation, mainly meniscal scaffold implantation (MSI), is suggested under this circumstance^8,9^. The MSI products are bioabsorbable structures with the biomechanical characteristics to protect the knee from excess loading and promote meniscus tissue regeneration^10^. Currently, the collagen meniscal implant (CMI)-Menaflex (Ivy Sports Medicine) and Actifit (Orteq)^11^ are the two types of meniscal scaffolds widely used.

While good postoperative pain relief and functional improvement are reported in Meniscal allograft transplantation (MAT)^12^ and MSI^11^, long-term survival rate of MAT is still concerning. Compared to a survival rate of 85% at 10 years in MSI^13^, that of MAT is reported to be 45–75% at 10 years^6,14^, and about 57% at 20 years^15^. Furthermore, problems like graft extrusion^16^, immunologic rejection^17^, the complicated technique^17^, the limited availability of allografts^18^, and graft size mismatching^19^, also disturb the surgeons to select the MAT.

To our knowledge, no study has compared the clinical outcomes of MAT and MSI. Although the indications of them are not exactly the same, it is necessary to compare their clinical outcome. Patients with partial meniscal defects face the choice of receiving MSI or not. Partial meniscal defects without MSI are more likely to cause subsequent meniscal injury and arthritis, ultimately leading to the requirement of MAT. So the option for these patients is actually either to receive MSI now or receive MAT in the future. Therefore, in this study, we evaluated the clinical outcomes of two techniques. We hypothesized that both MAT and MSI would lead to satisfactory clinical outcomes and MSI would have better patient-reported outcome measures (PROMs) and higher long-term survival rates.

## Methods

### Literature search and data sources

Following the PRISMA^20^, Supplemental Digital Content 1, http://links.lww.com/JS9/C505, Supplemental Digital Content 2, http://links.lww.com/JS9/C506 (Preferred Reporting Items for Systematic Reviews and Meta-Analyses) and AMSTAR^21^, Supplemental Digital Content 3, http://links.lww.com/JS9/C507 (Assessing the methodological quality of systematic reviews) guidelines, an extensive literature search was conducted to evaluate the outcomes of all meniscal transplants published before September 2023. The study protocol was registered at PROSPERO online (International prospective register of systematic reviews). The search was performed using the PubMed, Embase, and Cochrane Library electronic databases to identify all studies that reported the clinical outcomes of MAT or MSI. Keywords included menisc*, implant*, transplant*, scaffold, and allograft. Reference lists of related systematic reviews were also evaluated.

### Study selection

We reviewed, evaluated and selected the studies using the following inclusion criteria: (1) clinical studies of MAT or MSI, (2) all levels of evidence, and (3) English language. Non-human studies (animal experiments), review articles, cadaveric studies, case reports, and articles published in non-English languages were all excluded. If the same author published multiple studies on the clinical outcomes of MAT or MSI, only the last published article was included. Two reviewers independently screened the titles and abstracts of the searched studies and selected relevant studies for a full-text review. When it was difficult to decide whether to include or exclude an article based on its title and abstract, we would review the full text of the article for a decision. All references of enrolled studies were also examined. Study selection was based on the consensus of the reviewers. Disagreements were resolved by discussion with a third-party investigator.

### Data extraction

Each study that met the inclusion criteria was abstracted for information regarding the following: the year of publication, study type, level of evidence, mean age of the research participants at the time of surgery, mean follow-up duration, sample size, side, laterality, BMI, sex, preoperative and final follow-up PROMs, RTS rate, survival rate, and complications. The Lysholm score was selected as the primary outcome, which was the most commonly used PROM. The secondary outcomes included return to sports (RTS) rate, survival rate, complication rate, and other frequently used PROMs, including the Tegner activity score, the International Knee Documentation Committee (IKDC) subjective form, the visual analog scale (VAS) for pain, and the Knee injury and Osteoarthritis Outcome Score (KOOS). The backgrounds and explanations about the PROMs were listed in supplemental file 1, Supplemental Digital Content 4, http://links.lww.com/JS9/C508.

### Quality assessment

Based on study design, the quality of randomized controlled trials (RCTs) were evaluated using the Cochrane Risk of Bias assessment tool 2 (RoB2)^22^, while the quality of other included studies were assessed via the Coleman methodological Scale (CMS)^23^ with a range of 0–100. Two separate reviewers assessed study quality independently, with disagreements resolved by consensus with a third author.

### Data synthesis

Statistical analysis was performed using SPSS (IBM Corp. Released 2017. IBM SPSS Statistics for Macintosh, Version 25.0. IBM Corp.). Quantitative analysis was performed for each study. Since different studies reported different outcome scores, the scope of each weighted average score was stated separately^9^, including the corresponding number of studies and patients, respectively.

## Results

### Literature search results

**Figure 1:**
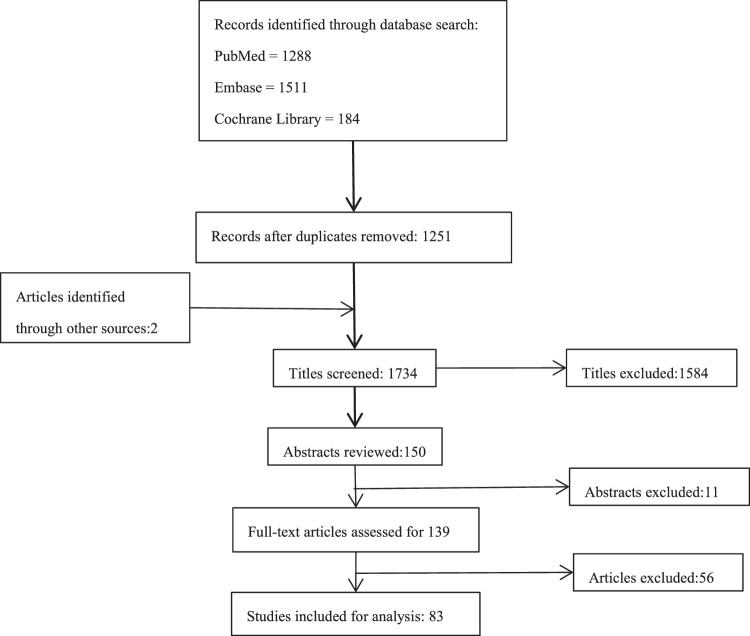
Systemic review algorithm using PRISMA guidelines. PRISMA, Preferred Reporting Items for Systematic Reviews and Meta-Analyses.

The study selection process was summarized by PRISMA flowchart in Figure 1. Through the literature search, we found a total of 2983 studies, including 1288 studies in PubMed (MEDLINE), 1511 studies in Embase, and 184 studies in the Cochrane Library. Two studies were found through the manual search, and 1251 duplicated studies were removed. After screening the titles and abstracts, and performing a full-text review on the remaining 139 studies, 83 studies were ultimately included in the present study, as shown in Table 1. The only RCT included was evaluated by the Cochrane RoB2. The overall risk of bias judgement was some concern. It showed low risk of bias in randomization process, deviations from intended interventions, and selection of the reported result. While the risk of bias in the measurement of the outcome was high due to different rehabilitation protocol. And the risk of bias in Missing Outcome Data was unclear. The CMS^23^ was calculated for all the other included studies, with an average total score of 57.08±9.10 (range, 23–76).

**Table 1:** Characteristics of the included studies.

Author	Year	*n*	Type of study	Level of evidence	Implant	CMS
Akkaya *et al*.^24^	2020	20	Case series	IV	Actifit	48
Baynat *et al*.^25^	2014	18	Prospective cohort	IV	Actifit	54
Bouyarmany *et al*.^26^	2014	54	Prospective cohort	IV	Actifit	51
Condelo *et al*.^27^	2021	67	Retrospective cohort	IV	Actifit	39
De Coninck *et al*.^28^	2013	26	Case series	IV	Actifit	61
Dhollander *et al*.^29^	2016	44	Case series	IV	Actifit	66
Efe *et al*.^30^	2012	10	Prospective cohort	IV	Actifit	60
Faivre *et al*.^31^	2015	20	Prospective cohort	IV	Actifit	59
Filardo *et al*.^32^	2017	16	Case series	IV	Actifit	59
Gelber *et al*.^33^	2021	62	Retrospective cohort	IV	Actifit	58
Haspl *et al*.^34^	2021	9	Retrospective cohort	IV	Actifit	41
Kon *et al*.^11^	2014	18	Case series	IV	Actifit	59
Leroy *et al*.^35^	2017	15	Prospective cohort	IV	Actifit	60
Monllau *et al*.^36^	2018	32	Case series	IV	Actifit	69
Olivos *et al*.^37^	2021	6	Case series	IV	Actifit	52
Schuttler *et al*.^38^	2015	16	Case series	IV	Actifit	58
Toanen *et al*.^39^	2020	114	Case series	IV	Actifit	23
Verdonk *et al*.^40^	2012	52	Case series	IV	Actifit	62
Bulgheroni *et al*.^17^	2010	34	Case series	IV	CMI	71
Hirschmann *et al*.^41^	2013	67	Prospective cohort	IV	CMI	61
Kovacs *et al*.^42^	2021	57	Retrospective cohort	IV	CMI	41
Linke *et al*.^43^	2006	60	Prospective cohort	IV	CMI	61
Monllau *et al*.^44^	2011	25	Therapeutic case series.	IV	CMI	64
Rodkey *et al*.^45^	2008	75	Randomized controlled trial	I	CMI	/
Rodkey *et al*.^46^	1999	8	Case series	IV	CMI	76
Schenk *et al*.^47^	2020	39	Case series	IV	CMI	51
Stone *et al*.^48^	1997	9	Prospective cohort	IV	CMI	63
Zaffagnini *et al*.^49^	2012	24	Prospective cohort	IV	CMI	64
Zaffagnini *et al*.^13^	2011	17	Case series	II	CMI	65
Bulgheroni *et al*.^10^	2016	25	Case series	IV	Actifit/CMI	66
Reale *et al*.^50^	2022	22	Prospective cohort	IV	Actifit/CMI	61
Spencer *et al*.^51^	2012	12	Case series	IV	Actifit/CMI	64
Abat *et al*.^52^	2012	33	Prospective cohort	II	allograft	56
Alentorn *et al*.^53^	2010	15	Retrospective cohort	IV	allograft	42
Baldairon *et al*.^54^	2019	5	Case series	IV	allograft	64
Cameron *et al*.^55^	1997	67	Retrospective cohort	IV	allograft	63
Chalmerss *et al*.^56^	2013	13	Retrospective cohort	IV	allograft	49
Chang *et al*.^57^	2008	12	Case series	III	allograft	64
Cvetanovich *et al*.^58^	2020	87	Case series	IV	allograft	42
Cole *et al*.^12^	2006	40	Prospective cohort	IV	allograft	65
Farr *et al*.^59^	2007	36	Prospective cohort	IV	allograft	61
Fukushima *et al*.^60^	2004	40	Case series	IV	allograft	53
Ha *et al*.^61^	2011	22	Case series	IV	allograft	53
Hommen *et al*.^62^	2007	22	Retrospective cohort	IV	allograft	54
Jang *et al*.^63^	2015	13	Retrospective cohort	IV	allograft	65
Kazi *et al*.^64^	2015	86	Retrospective cohort	IV	allograft	49
Kempshall *et al*.^65^	2015	60	Prospective cohort	III	allograft	49
Kim *et al*.^66^	2018	30	Case series	IV	allograft	59
Kim *et al*.^67^	2020	249	Retrospective cohort	III	allograft	49
Kocher *et al*.^68^	2016	7	Case series	IV	allograft	56
Koh *et al*.^69^	2018	37	Retrospective cohort	IV	allograft	46
LaPrade *et al*.^70^	2010	34	Prospective cohort	IV	allograft	63
Lee *et al*.^71^	2017	87	Retrospective cohort	IV	allograft	64
Liu *et al*.^72^	2020	22	Case series	IV	allograft	65
Marcacci *et al*.^73^	2012	32	Prospective cohort	IV	allograft	61
Marcacci *et al*.^74^	2014	12	Case series	IV	allograft	67
McCormick *et al*.^75^	2014	172	Retrospective cohort	IV	allograft	67
Milachowski *et al*.^76^	1989	22	Case series	IV	allograft	59
Noyes *et al*.^6^	2016	72	Prospective cohort	IV	allograft	49
Noyes *et al*.^77^	2005	38	Case series	IV	allograft	66
Parkinson *et al*.^78^	2016	71	Prospective cohort	III	allograft	49
Potter *et al*.^79^	1996	29	Case series	IV	allograft	59
Puzzitiello *et al*.^80^	2020	17	Case series	IV	allograft	49
Rath *et al*.^81^	2001	27	Prospective cohort	IV	allograft	59
Roumazeille *et al*.^82^	2015	22	Retrospective cohort	IV	allograft	48
Rue *et al*.^83^	2008	31	Case series	IV	allograft	59
Ryu *et al*.^84^	2002	26	Retrospective cohort	IV	allograft	49
Saltzman *et al*.^85^	2017	40	Prospective cohort	IV	allograft	41
Searle *et al* ^86^	2020	43	Case series	IV	allograft	59
Sekiya *et al*.^87^	2003	28	Retrospective cohort	IV	allograft	59
Stone *et al*.^88^	2015	49	Prospective cohort	IV	allograft	58
van Arkel *et al*.^89^	2002	63	Prospective cohort	IV	allograft	65
Van Der Straeten *et al*.^90^	2016	329	Retrospective cohort	IV	allograft	64
van der wal *et al*.^91^	2020	111	Prospective cohort	III	allograft	45
verdonk *et al*.^14^	2005	61	Prospective cohort	IV	allograft	52
Vundelinckx *et al*.^92^	2014	30	Retrospective cohort	IV	allograft	63
Waterman *et al*.^93^	2016	230	Retrospective cohort	IV	allograft	41
Wirth *et al*.^94^	2002	23	Prospective cohort	IV	allograft	61
Yoldas *et al*.^95^	2003	12	Retrospective cohort	IV	allograft	51
Yoon *et al*.^19^	2014	30	Retrospective cohort	IV	allograft	68
Yoon *et al*.^19^	2014	56	Retrospective cohort	III	allograft	69
Zaffagnini *et al*.^96^	2016	147	Retrospective cohort	IV	allograft	56
Zhang *et al*.^97^	2012	19	Prospective cohort	IV	allograft	52

CMS, Coleman Methodological Score.

### Demographics

In the final cohort (83 studies, 3932 patients), the overall weighted mean follow-up was 64.50±47.38 month in MAT and 57.54±34.22 month in MSI. The MAT group had a mean weighted age of 33.90±13.30 years and 66.04% (1748 in 2647) were male. Whereas the MSI group had a mean weighted age of 36.00±11.52 years and the proportion of males reached 73.01% (717 in 982). The overall weighted mean BMI was 25.09±4.47 in MAT group and 25.28±4.19 in MSI group. The Mean time from meniscectomy was 9.33±6.64 years for MAT and 6.22±5.66 years for MSI. There was no significant difference in the distribution ratio of left and right knees between the two groups (*P*=0.123). MAT was more performed in the lateral meniscus (*P*<0.001) while MSI was more performed in the medial (*P*<0.001). The demographics are shown in Table 2.

**Table 2:** Demographics of MAT and MSI.

	MAT	*n* (papers)	*n* (patients)	MSI	*n* (papers)	*n* (patients)	*P*
Age (year)	33.90±10.30	39	2764	36.00±11.52	27	941	<0.001
Sex, *n* (%)		33	2647		26	982	
Male	1748 (66.04)			717 (73.01)			
Female	899 (33.96)			265 (26.99)			
BMI (kg/m^2^)	25.09±4.47	10	940	25.28±4.19	10	225	=0.562
Follow-up (month)	64.50±47.38	37	2318	57.54±34.22	13	518	=0.002
<24	5			9			
24<FU<60	23			8			
60<FU<120	6			3			
120<FU	5			3			
Mean time from meniscectomy (year)	9.33±6.64	19	1191	6.22±5.66	4	119	<0.001
Side, *n* (%)		10	1274		9	265	=0.123
Left	595 (46.70)			110 (41.51)			
Right	679 (53.30)			155 (58.49)			
Medial/lateral, *n* (%)		29	2049		20	692	<0.001
Medial	877 (42.80)			479 (69.22)			
Lateral	1172 (57.20)			213 (30.78)			

*P* values are listed for analysis of *t*-tests or χ2 tests for comparisons between MAT and MSI.

FU, follow-up; MAT, meniscal allograft transplantation; MSI, meniscal scaffold implantation.

### Patient-report outcomes (PROMs)

The Lysholm score, most commonly used in the included studies, was selected as the primary outcome in this study. Besides, the Tegner activity score, the International Knee Documentation Committee (IKDC) subjective form, the visual analog scale (VAS) for pain, and the Knee injury and Osteoarthritis Outcome Score (KOOS), also frequently used in MAT and MSI, were determined as the secondary outcomes.

There were statistically significant improvements in all these PROMs from preoperative values to final follow-up (*P*<0.05) in both groups (Table 3). The mean Lysholm score improved from 55.65±17.21 to 82.12±15.36 (*P*<0.001) in MAT and from 59.62±19.67 to 86.57±15.28 (*P*<0.001) in MSI. The mean VAS pain scale improved from 5.69±2.55 to 1.99±2.01 (*P*<0.001) in MAT and from 5.34±2.45 to 2.34±2.26 (*P*<0.001) in MSI. The Tegner activity score improved from 2.83±2.03 to 4.62±1.93 (*P*<0.001) in MAT, and from 3.18±1.58 to 4.71±1.85 (*P*<0.001) in MSI. IKDC Subjective Score improved from 48.62±15.34 to 71.26±15.30 (*P*<0.001) in MAT and from 41.61±17.15 to 69.38±20.25 (*P*<0.001) in MSI. Similarly, the weighted average overall KOOS score for pain, symptom, sports, ADL, and QOL all increased significantly (*P*<0.001) at the last follow-up.

**Table 3:** Preoperative and final follow-up PROMs.

	MAT				MSI			
Knee Scoring Scale	*N* (studies)	*N* (patients)	Preoperative	Final follow-up	*N* (studies)	*N* (patients)	Preoperative	Final follow-up
Lysholm	20	1191	56.65±17.21	82.12±15.36***	13	475	59.62±19.67	86.57±15.28***
VAS pain	9	526	5.69±2.55	1.99±2.01***	16	624	5.34±2.45	2.34±2.26***
Tegner	13	725	2.83±2.03	4.62±1.93***	9	260	3.18±1.58	4.71±1.85***
IKDC	13	812	48.62±15.34	71.26±15.30***	12	547	41.61±17.15	69.38±20.25***
KOOS	7	279			9	351		
Symptoms		279	55.85±13.71	70.71±13.22***		351	58.77±19.38	77.11±18.41***
Pain		279	56.41±13.62	77.62±13.93***		351	54.40±19.82	78.59±20.66***
ADL		279	68.58±14.81	85.12±11.92***		351	62.14±21.95	82.93±20.33***
QOL		279	28.21±17.27	51.72±18.47***		351	32.08±16.63	57.00±24.31***
Sport		279	28.99±17.53	53.68±19.86***		351	29.39±24.39	56.00±30.27***

ADL, activities of daily living; IKDC, International Knee Documentation Committee; KOOS, Knee Injury and Osteoarthritis Outcome Score; MAT, meniscal allograft transplantation; MSI, meniscal scaffold implantation; PROM, patient-reported outcome measure; QOL, quality of life; VAS, visual analog scale.

“*” are listed for significant difference between preoperative scores and postoperative scores.

*** *P*<0.001.

The preoperative and postoperative PROMs were shown in Table 4. MSI had higher preoperative Tegner activity score (*P*=0.012), KOOS symptoms (*P*=0.034), and KOOS QOL (*P*=0.004); similar KOOS pain (*P*=0.149) and KOOS sport (*P*=0.818); and lower IKDC (*P*<0.001) and KOOS ADL (*P*<0.001) than MAT. At final follow-up, MSI had higher Lysholm score (*P*<0.001), KOOS symptoms (*P*<0.001), and KOOS QOL (*P*=0.003) than MAT. Interestingly, the VAS pain in MSI was less preoperatively (*P*=0.018) and higher postoperatively (*P*=0.006) than that in MAT.

**Table 4:** Comparison of preoperative and final follow-up PROMs between MAT and MSI.

	Preoperative					Postoperative				
Knee Scoring Scale	*N* (patients)	MAT	*N* (patients)	MSI	*P*	*N* (patients)	MAT	*N* (patients)	MSI	*P*
Lysholm	1191	56.65±17.21	475	59.62±19.67	0.002	1191	82.12±15.36	475	86.57±15.28	<0.001
VAS pain	526	5.69±2.55	624	5.34±2.45	0.018	526	1.99±2.01	624	2.34±2.26	0.006
Tegner	725	2.83±2.03	260	3.18±1.58	0.012	725	4.62±1.93	260	4.71±1.85	0.514
IKDC	812	48.62±15.34	547	41.61±17.15	<0.001	812	71.26±15.30	547	69.38±20.25	0.052
KOOS	279		351			279		351		
Symptoms	279	55.85±13.71	351	58.77±19.38	0.034	279	70.71±13.22	351	77.11±18.41	<0.001
Pain	279	56.41±13.62	351	54.40±19.82	0.149	279	77.62±13.93	351	78.59±20.66	0.502
ADL	279	68.58±14.81	351	62.14±21.95	<0.001	279	85.12±11.92	351	82.93±20.33	0.111
QOL	279	28.21±17.27	351	32.08±16.63	0.004	279	51.72±18.47	351	57.00±24.31	0.003
Sport	279	28.99±17.53	351	29.39±24.39	0.818	279	53.68±19.86	351	56.00±30.27	0.270

ADL, activities of daily living; IKDC, International Knee Documentation Committee; KOOS, Knee Injury and Osteoarthritis Outcome Score; MAT, meniscal allograft transplantation; MSI, meniscal scaffold implantation; PROM, patient-reported outcome measure; QOL, quality of life; VAS, visual analog scale.

The mean improvements of these PROMs from preoperative to final follow-up were shown in Table 5. The PROMs improvements in MSI are similar of Lysholm score (*P*=0.105) and Tegner activity score (*P*=0.061), more of IKDC (*P*<0.001), KOOS pain (*P*=0.036), KOOS symptom (*P*=0.010), and KOOS ADL (*P*=0.004) scores, and less of VAS pain (*P*<0.001) than those in MAT.

**Table 5:** Mean improvements of PROMs.

	MAT			MSI			
Knee Scoring Scale	*N* (studies)	*N* (patients)	Mean improvement	*N* (studies)	*N* (patients)	Mean improvement	*P*
Lysholm	20	1191	25.47±16.36	13	475	26.95±17.88	0.105
VAS pain	9	526	3.70±2.33	16	624	3.00±2.36	<0.001
Tegner	13	725	1.79±2.80	9	260	1.53±2.43	0.061
IKDC	13	812	22.64±15.32	12	547	27.77±18.89	<0.001
KOOS	7	279		9	351		
Symptoms		279	14.86±13.47		351	18.34±18.91	0.010
Pain		279	21.21±13.78		351	24.19±20.25	0.036
ADL		279	16.54±13.60		351	20.79±21.19	0.004
QOL		279	23.51±17.90		351	24.92±21.52	0.380
Sport		279	24.69±18.80		351	26.61±27.80	0.324

*P* values are listed for analysis of *t*-tests for comparisons of mean improvements between MAT and MSI.

ADL, activities of daily living; IKDC, International Knee Documentation Committee; KOOS, Knee Injury and Osteoarthritis Outcome Score; MAT, meniscal allograft transplantation; MSI, meniscal scaffold implantation; PROM, patient-reported outcome measure; QOL, quality of life; VAS, visual analog scale.

### Return to sports (RTS)

The RTS rate (Table 6) was reported in 12 studies of MAT^85,96^ and 2 studies of MSI^24,25^. The mean weighted RTS rate of 95.10% (range 91.30–100%) in MSI was significantly higher (*P*=0,002) than 73.50% (range 28.60–91.70%) in MAT. The mean weighted return to pre-injury level rate of 48.26% (range 7.00–100%) in MAT was similar with 50.00% in MSI. The RTS time ranged between 7.6 and 16.9 months in MAT, while 18 MSI studies mentioned a return to full activity, sports, or unrestricted activities of daily living at 6 months post-surgery^10,17,28,98^.

**Table 6:** RTS rate and RTS time.

	*n* (patients)	MAT	*n* (patients)	MSI	*P*
RTS rate (%)	432	73.50%	41	95.10%	0.002
RTS time (month)	221	11.46±6.19		NR	
Return to pre-injury activity level rate (%)	292	48.26%	18	50.00%	0.888

MAT, meniscal allograft transplantation; MSI, meniscal scaffold implantation; NR, not reported; RTS, return to sports.

### Survival

The 5-year survival rates, reported in 15 MAT studies^85,89,99,100^ and 4 MSI studies^13,29,39,45^, were similar (*P*=0.883) in average between 84.92% (range 62.00–98.00%) in MAT and 84.80% (range 62.20–89.00%) in MSI. The 10-year survival rates, reported in 8 MAT studies^6,92,101^ and 2 MSI studies^13,50^, were higher (*P*=0.006) in MSI (81.85%) than that of MAT (66.57%). The 15-year survival rates were reported in 4 MAT studies averaged as 51.07%, but unavailable in MSI. The averaged K-M survival time, reported in 5 MAT studies^14,85,89,90,92^, was 13.74 years. The survival rates and K–M survival time were summarized in Table 7.

**Table 7:** Survival rate and K–M survival time.

	*n* (studies)	*n* (patients)	MAT	*n* (studies)	*n* (patients)	MSI	*P*
Failure rate	25	1903	12.78%	14	566	13.20%	0.764
Time to failure (year)	8	507	6.21		NR		
5-year survival rate	15	1103	85.37%	4	335	84.80%	0.777
10-year survival rate	8	451	66.57%	2	64	80.37%	0.034
15-year survival rate	4	151	51.07%		NR		
K–M survival time (year)	5	652	13.74		NR		
Complication rate	26	1177	19.82%	13	555	7.85%	0.001

*P* values are listed for analysis of χ2 tests for comparisons of survival rates between MAT and MSI.

MAT, meniscal allograft transplantation; MSI, meniscal scaffold implantation; NR, not reported.

However, the definitions of survival were different among studies. MAT studies had various self-defined endpoint definitions combining outcome scores, MRI criteria, and surgical failures, while MSI defined any associated reoperations as failure. Therefore, if the endpoint with any associated reoperations were used, the mean 5-year survival rates, reported in 7 MAT studies^64,75,78,85,92,99,100^ and 4 MSI studies^13,29,39,45^, were similar (*P*=0.093) in MAT (85.03%) and MSI (84.80%). The 10-year survival rates, reported in 5 MAT studies^62,64,92,99,100^ and 2 MSI studies^13,50^, were higher (*P*<0.001) in MSI (81.85%) than that in MAT (68.81%).

### Complication

The overall complication rates of 1050 cases in 26 MAT studies^57,60,68,73,89,94,102^ was 17.52%, and higher (*P*<0.001) than 9.64% of 529 cases in 13 MSI studies. Local and general complications of MAT and MSI were summarized and shown in Table 8.

**Table 8:** Local and general complications of MAT and MSI.

	MAT (*n*=1050)		MSI (*n*=529)		
	*n*	Rate	*n*	Rate	*P*
Local complications					
Graft retear	58	5.52%	5	0.95%	<0.001
Graft extrusion	22	2.10%	2	0.38%	0.008
Graft shrinkage	3	0.29%	NR		
Cartilage lesion	NR		1	0.19%	
Subchondral edema	NR		1	0.19%	
Synovitis	2	0.19%	5	0.95%	0.033
Arthrofibrosis	31	2.95%	4	0.76%	0.005
Joint instability	NR		1	0.19%	
Swelling/redness/effusion	10	0.95%	11	2.08%	0.065
Joint pain	20	1.90%	7	1.32%	0.400
Mechanical symptoms	4	0.38%	2	0.38%	0.993
General complications					
Quadriceps weakness	NR		1	0.19%	
Patellofemoral symptoms	NR		1	0.19%	
Hemarthrosis	1	0.10%	1	0.19%	0.621
Nerve damage	6	0.57%	4	0.76%	0.652
Infection	23	2.19%	3	0.57%	0.019
Thrombosis	4	0.38%	2	0.38%	0.993
Overall complication rate	184	17.52%	51	9.64%	<0.001

*P* values are listed for analysis of χ2 tests for comparisons of complication rates between MAT and MSI.

MAT, meniscal allograft transplantation; MSI, meniscal scaffold implantation; NR, not reported.

## Discussion

The main purpose of this review was to evaluate and compare the postoperative outcomes of MAT and MSI to support the clinical treatment decision for partial meniscal defect. Patients with unrepairable partial meniscus injury were traditionally treated with meniscoplasty. Postoperative changes in meniscus morphology and mechanical structure might lead to further degeneration of meniscus, and that’s when MAT is required. Since the efficacy of MAT was not satisfying, we consider whether MSI can be performed for patients with partial meniscal defect to avoid further meniscal degeneration and the possibility of MAT.

We compared the PROMs, RTS, and survival rate. All the PROMs, including Lysholm score, showed significant improvements in both MAT and MSI. The RTS rate was 73.50% in MAT and 95.10% in MSI (*P*=0.002). The overall 10-year survival rate was 66.57% in MAT and 80.37% in MSI (*P*=0.034). The overall complication rate was 19.82% in MAT and 7.85% in MSI (*P*=0.001).

Lysholm score was significantly improved after the surgery in both groups, indicating that both MAT and MSI significantly improved knee function. Both preoperative (*P*=0.002) and postoperative (*P*<0.001) Lysholm score were significantly higher in MSI group than MAT, but there was no significant difference in the mean improvement (*P*=0.105). This indicated that MAT patients had worse knee function both preoperatively and postoperatively, but there was no significant difference in the efficacy between MSI and MAT.

With evaluation using VAS, Tegner, IKDC, and KOOS, both MAT and MSI were noticed to bring significant benefit on pain relief, activity level, knee function, and quality of life. The preoperative and postoperative PROMs were compared between the two groups. MSI had significantly higher preoperative Tegner (*P*=0.012), VAS pain (*P*=0.018), KOOS symptoms (*P*=0.034), KOOS QOL (*P*=0.004), similar KOOS pain (*P*=0.149) and KOOS sport (*P*=0.818), and lower IKDC (*P*<0.001) and KOOS ADL (*P*<0.001) than MAT. At final follow-up, KOOS symptoms (*P*<0.001) and KOOS QOL (*P*=0.003) in MSI were significantly better than MAT. In terms of pain, MAT had better VAS pain (*P*=0.006) and similar KOOS pain (*P*=0.502) compared with MSI. No significant difference was found in IKDC (*P*=0.052), Tegner (*P*=0.514), KOOS ADL (*P*=0.111) and KOOS sport (*P*=0.270). Comparing the mean improvements, Tegner activity score (*P*=0.061), KOOS sports (*P*=0.324), and KOOS QOL (*P*=0.380) showed no significant difference between MAT and MSI, while IKDC (*P*<0.001), KOOS symptom (*P*=0.010), KOOS pain (*P*=0.036), and KOOS ADL (*P*=0.004) were significantly better in MSI. VAS pain (*P*<0.001) was significantly better in MAT. Although the primary outcome showed that MSI had better preoperative and postoperative knee function and there was no difference in function improvement between the two groups, some secondary outcomes showed different results. IKDC showed that preoperative knee function was better in MAT, and significantly more improved in MSI. VAS pain showed that MAT had better pain reliving effect.

Although most of MAT and MSI studies reported returning to low-intensity sports after surgery, it was very difficult to compare the outcome of RTS between two groups. The RTS rate were reported in 9 MAT studies (range 28.60–92.00%), and 2 MSI studies (range 91.30–100%)^24,25^. However, the included studies reported athletes in military^93^, different sports^53,56,74,96^, and people with different sports levels^85^, which leaded to difficulty in comparison. Besides, a measurement called Tegner index^50^ was brought in to compare the activity level between the two groups, which represents the percentage of the lost activity level that was regained as a result of the treatment intervention, and no difference was found between the two groups.

Compared with MAT, MSI had higher 10-year survival rate (*P*=0.034), and similar 5-year survival rate with MAT (*P*=0.964). However, the definitions of the survival endpoint were different among the studies. It was generally defined as any reoperation associated with the index surgery, but in some MAT studies, various combined definitions including reoperations, PROMs, and MRI results were used. Therefore, the survival rates with endpoint defined as any associated reoperation only were analyzed. The 5-year survival rate was 85.03% in MAT and 84.80% in MSI, with no significant difference (*P*=0.093). The 10-year survival rate was 68.81% in MAT and 80.37% in MSI, with significant difference (*P*<0.001). In addition, it was necessary to consider not only which had higher survival rate but also what we can do after the survival endpoint. While MAT patients were usually treated with joint replacement or another MAT^42^, MSI patients had more options. It was reported that when the MSI graft reaches the endpoint, MAT can be performed in about one-third of them, or the scaffolds can be removed directly to retain the rest meniscus tissue, and of course joint replacement can be done^39^.

The mean overall complication rate was 17.52% in MAT, and 9.64% in MSI in this review (*P*<0.001). Main surgical complications after MAT included: effusions, synovial reaction, infection, loss of motion, technical failures, graft shrinkage and graft extrusion^103^, and that in MSI generally include swelling, pain, nerve injury, instability, infection, deep venous thrombosis (DVT), wound problems, patellofemoral symptoms, fever, chronic synovitis and graft failure^104^. The most common complication was graft tear (5.52%) in MAT and swelling, redness, or effusion (2.08%) in MSI. Some of these complications were thought to be due to concomitant surgery, while others were not, such as meniscal allograft partial tears, arthrofibrosis, and infection^105^. According to Vundelinckx *et al*.^92^, the overall complication rate in MAT with or without concomitant procedure is estimated at 21.30%, while the percentage drops to 5.70% in isolated MAT. Besides, some studies reported symptoms with no clear cause as complications, such as joint pain and swelling, which indicated complications with these symptoms might have higher rates than reported, but this didn’t affect the overall complication rate.

Several concerns also existed in our research. Firstly, these two techniques involved different patient populations. According to this systematic review, the average age of patients who received MSI was significantly older than that of MAT. The mean time from meniscectomy was 9.33±6.64 years for MAT and 6.22±5.66 years for MSI, the difference was significant (*P*<0.001). And MAT was more performed in the lateral meniscus while MSI was more performed in the medial, and the difference between MAT and MSI was statistically significant (*P*<0.001).

Second, the indications were different between MAT and MSI. The indications were similar in the literatures, but there were still many disputes on the details. MAT was typically performed after total or subtotal meniscectomy, whereas MSI was indicated only for partial meniscal defects, since they require an intact meniscal rim and the presence of both anterior and posterior horns^9,106^. But it is recently reported that MAT can also be performed on partial meniscal defect patients, directly or after total meniscectomy, according to Seiter *et al*.^107^, despite long-term clinical results are still under-researched. In addition, the indication of MAT also usually includes: young patients, neutral alignment, stable cruciate and collateral ligaments, unicompartmental activity-related pain and effusion, and no signs of significant arthritic changes in the affected compartment^108^. However, in some studies, the indications have been expanded. Stone^109^ et al reported the results of 119 primary MATs in older patients (average age, 46.9 years; range, 14.1–73.2 years); in that study, 46.1% of the patients were older than 50 years. The same research group also reported 47 MATs performed in 45 patients with preoperative evidence of arthrosis and Outerbridge degeneration grade II (81% grade IV), with an average time to failure of 4.4 years^109^. MSI is repeatedly reported to be indicated for acute or irreparable injury or previous subtotal loss of meniscus^43,110,111^. However, in the RCT study reported by Rodkey *et al*.^45^ in 2008, CMI was not found to have any benefit for patients with an acute injury compared with partial meniscectomy regarding the reoperation rate, survival rate, VAS pain score, Lysholm score, and Patient self-assessment score. And new tissue ingrowth and new matrix production were found in 97% of the patients in the chronic group and 70% of the patients in the acute group, which also supports the view that CMI seems more effective in chronic meniscal injury. This suggests that indications for both procedures are evolving, and that researchers continue to explore them, even though there is a general consensus on the appropriate indications.

Third, the follow-up time of MAT group was significantly longer, which confirmed the view that MAT-related long-term studies were more mature than MSI. Nevertheless, due to the limited information on MAT’s long-term cartilage protection, long-term investigations of both MAT and MSI with adequate cohort sizes are of necessity.

The present review has some limitations. When calculating the survival rates, we included all the studies with an average follow-up of more than the prescribed number of years, including studies in which patients with longer follow-up could not be discerned from those with less than prescribed years of follow-up. This might lead to the inclusion criteria bias. In addition, High-quality long-term studies are not sufficient for MSI, especially for Actifit. According to Reale *et al*.^9^, there was no significant difference in survival rate between CMI and Actifit during long-term follow-up, so we did not distinguish between CMI and Actifit in the calculation of long-term survival rate. Besides, although up to 83 studies were included, the level of evidence was low, with only one RCT. These limitations might lead to slightly less solidity in the results. More high-quality evidences on long-term survival for MSI are needed in the future, especially for Actifit.

## Conclusion

This systematic review drew the following conclusions: First, both MAT and MSI had significant efficacy, and all PROMs were significantly improved after surgery. Second, the primary outcome showed that MSI had better preoperative and postoperative knee function, and MAT and MSI were similarly effective. Third, the long-term survival rate of MSI is better than MAT, and the complications of MSI are less than MAT. Accordingly, both MAT and MSI were effective, and early MSI was encouraged to avoid MAT in partial meniscal defects.

## Ethical approval

It’s a systematic review.

## Consent

It’s a systematic review.

## Sources of funding

This work was supported by the National Natural Science Foundation of China [grant number 81972062]; the National Natural Science Foundation of China [grant number 82172509]; and the Shanghai Sailing Program [grant number 23YF1434300].

## Author contribution

J.D. and M.H. contributed to the study conception and design. All authors collected the data and performed the data analysis. All authors contributed to the interpretation of the data and the completion of figures and tables. All authors contributed to the drafting of the article and final approval of the submitted version.

## Conflicts of interest disclosure

The authors declare that there is no conflict of interests regarding the publication of this paper.

## Research registration unique identifying number (UIN)

CRD42023458934.

## Guarantor

Jize Dong and Jiwu Chen.

## Data availability statement

The datasets used and analyzed during the current study are publicly available.

## Provenance and peer review

Not commissioned, externally peer-reviewed.

## Supplementary Material
