# Supplementary material for: Outcome comparison of meniscal allograft transplantation (MAT) and meniscal scaffold implantation (MSI): a systematic review
Source: Int J Surg. 2024 May 13;110(8):5112–23. doi: 10.1097/JS9.0000000000001587 (PMC11325955; doi:10.1097/JS9.0000000000001587)
Supplement: Supplementary file 1 [file js9-110-5112-s001.docx]

| **Section and Topic** | **Item #** | **Checklist item** | **Location where item is reported** |
| --- | --- | --- | --- |
| **TITLE** | | |  |
| Title | 1 | The report is identified as a systematic review. | 1 |
| **ABSTRACT** | | |  |
| Abstract | 2 | The structured abstract include background, purpose, methods, results, and conclusion. | 2 |
| **INTRODUCTION** | | |  |
| Rationale | 3 | Described in the Introduction. | 3 |
| Objectives | 4 | Stated in the Introduction. | 4 |
| **METHODS** | | |  |
| Eligibility criteria | 5 | Stated in the Method. | 5 |
| Information sources | 6 | Described in the Method. | 6 |
| Search strategy | 7 | Described in the Method. | 7 |
| Selection process | 8 | Described in the Method. | 8 |
| Data collection process | 9 | Described in the Method. | 9 |
| Data items | 10a | Described in the Method. | 10a |
|  | 10b | Described in the Method. | 10b |
| Study risk of bias assessment | 11 | Described in the Method. | 11 |
| Effect measures | 12 | Described in the Method. | 12 |
| Synthesis methods | 13a | Described in the Method. | 13a |
|  | 13b | Described in the Method. | 13b |
|  | 13c | Described in the Method. | 13c |
|  | 13d | Described in the Method. | 13d |
|  | 13e | Described in the Method. | 13e |
|  | 13f | Described in the Method. | 13f |
| Reporting bias assessment | 14 | Described in the Method. | 14 |
| Certainty assessment | 15 | Described in the Method. | 15 |
| **RESULTS** | | |  |
| Study selection | 16a | Described in the Results. | 16a |
|  | 16b | Described in the Results. | 16b |
| Study characteristics | 17 | Described in the Results. | 17 |
| Risk of bias in studies | 18 | Described in the Results. | 18 |
| Results of individual studies | 19 | Described in the Results. | 19 |
| Results of syntheses | 20a | Described in the Results. | 20a |
|  | 20b | Described in the Results. | 20b |
|  | 20c | Described in the Results. | 20c |
|  | 20d | Described in the Results. | 20d |
| Reporting biases | 21 | Described in the Results. | 21 |
| Certainty of evidence | 22 | Described in the Results. | 22 |
| **DISCUSSION** | | |  |
| Discussion | 23a | Described in the Discussion. | 23a |
|  | 23b | Described in the Discussion. | 23b |
|  | 23c | Described in the Discussion. | 23c |
|  | 23d | Described in the Discussion. | 23d |
| **OTHER INFORMATION** | | |  |
| Registration and protocol | 24a | Registration information is provided. | 24a |
|  | 24b | The review protocol can be accessed in PROSPERO. | 24b |
|  | 24c | none | 24c |
| Support | 25 | Described in the Title Page and the Author Disclosure. | 25 |
| Competing interests | 26 | Described in the Conflict of Interest of the Title Page. | 26 |
| Availability of data, code and other materials | 27 | Described in the Data Statement. | 27 |

*From:*  Page MJ, McKenzie JE, Bossuyt PM, Boutron I, Hoffmann TC, Mulrow CD, et al. The PRISMA 2020 statement: an updated guideline for reporting systematic reviews. BMJ 2021;372:n71. doi: 10.1136/bmj.n71

For more information, visit: <http://www.prisma-statement.org/>
