# Supplementary material for: Outcome comparison of meniscal allograft transplantation (MAT) and meniscal scaffold implantation (MSI): a systematic review
Source: Int J Surg. 2024 May 13;110(8):5112–23. doi: 10.1097/JS9.0000000000001587 (PMC11325955; doi:10.1097/JS9.0000000000001587)
Supplement: Supplementary file 2 [file js9-110-5112-s002.pdf]

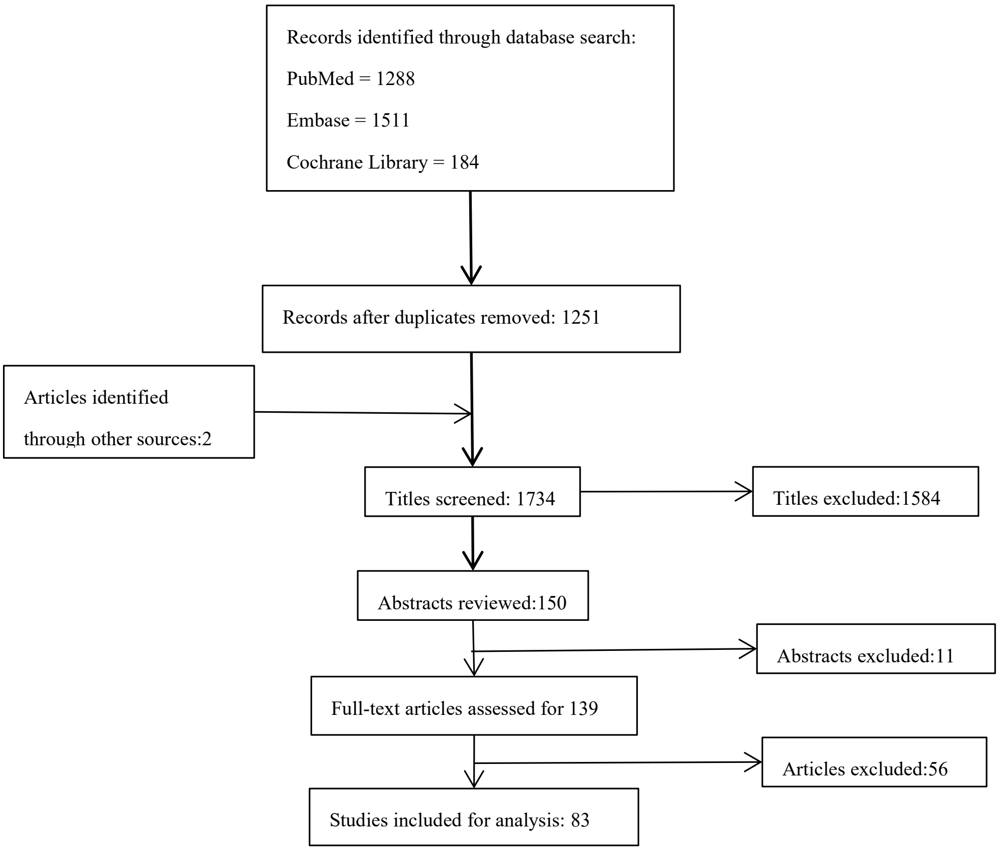

Figure 1. Systemic review algorithm using Preferred Reporting Items for Systematic Reviews and Meta-Analyses (PRISMA) guidelines.
