# Supplementary material for: Outcome comparison of meniscal allograft transplantation (MAT) and meniscal scaffold implantation (MSI): a systematic review
Source: Int J Surg. 2024 May 13;110(8):5112–23. doi: 10.1097/JS9.0000000000001587 (PMC11325955; doi:10.1097/JS9.0000000000001587)
Supplement: Supplementary file 4 [file js9-110-5112-s004.docx]

Table 1: Characteristics of the included studies

| Author | year | | n | | type of study | | level of evidence | | implant | | CMS | |
| --- | --- | --- | --- | --- | --- | --- | --- | --- | --- | --- | --- | --- |
| Akkaya et al. | 2020 | | 20 | | case series | | IV | | Actifit | | 48 | |
| Baynat et al. | 2014 | | 18 | | prospective cohort | | IV | | Actifit | | 54 | |
| Bouyarmany et al.^[1]^ | 2014 | | 54 | | prospective cohort | | IV | | Actifit | | 51 | |
| Condelo et al.^[2]^ | 2021 | | 67 | | retrospective cohort | | IV | | Actifit | | 39 | |
| De Coninck et al. | 2013 | | 26 | | case series | | IV | | Actifit | | 61 | |
| Dhollander et al. | 2016 | | 44 | | case series | | IV | | Actifit | | 66 | |
| Efe et al.^[3]^ | 2012 | | 10 | | prospective cohort | | IV | | Actifit | | 60 | |
| Faivre et al.^[4]^ | 2015 | | 20 | | prospective cohort | | IV | | Actifit | | 59 | |
| Filardo et al.^[5]^ | 2017 | | 16 | | case series | | IV | | Actifit | | 59 | |
| Gelber et al.^[6]^ | 2021 | | 62 | | retrospective cohort | | IV | | Actifit | | 58 | |
| Haspl et al.^[7]^ | 2021 | | 9 | | retrospective cohort | | IV | | Actifit | | 41 | |
| Kon et al. | 2014 | | 18 | | case series | | IV | | Actifit | | 59 | |
| Leroy et al.^[8]^ | 2017 | | 15 | | prospective cohort | | IV | | Actifit | | 60 | |
| Monllau et al.^[9]^ | 2018 | | 32 | | case series | | IV | | Actifit | | 69 | |
| Olivos et al.^[10]^ | 2021 | | 6 | | case series | | IV | | Actifit | | 52 | |
| Schuttler et al.^[11]^ | 2015 | | 16 | | case series | | IV | | Actifit | | 58 | |
| Toanen et al. | 2020 | | 114 | | case series | | IV | | Actifit | | 23 | |
| Verdonk et al.^[12]^ | 2012 | | 52 | | case series | | IV | | Actifit | | 62 | |
| Bulgheroni et al. | 2010 | | 34 | | case series | | IV | | CMI | | 71 | |
| Hirschmann et al.^[13]^ | 2013 | | 67 | | prospective cohort | | IV | | CMI | | 61 | |
| Kovacs et al.^[14]^ | 2021 | | 57 | | retrospective cohort | | IV | | CMI | | 41 | |
| Linke et al.^[15]^ | 2006 | | 60 | | prospective cohort | | IV | | CMI | | 61 | |
| Monllau et al.^[16]^ | 2011 | | 25 | | therapeutic case series. | | IV | | CMI | | 64 | |
| Rodkey et al. | 2008 | | 75 | | randomized controlled  trial | | I | | CMI | | / | |
| Rodkey et al.^[17]^ | 1999 | | 8 | | case series | | IV | | CMI | | 76 | |
| Schenk et al.^[18]^ | 2020 | | 39 | | case series | | IV | | CMI | | 51 | |
| Stone et al.^[19]^ | 1997 | | 9 | | Prospective cohort | | IV | | CMI | | 63 | |
| Zaffagnini et al.^[20]^ | 2012 | | 24 | | Prospective cohort | | IV | | CMI | | 64 | |
| Zaffagnini et al. | 2011 | | 17 | | case series | | II | | CMI | | 65 | |
| Bulgheroni et al. | 2016 | | 25 | | case series | | IV | | Actifit/CMI | | 66 | |
| Reale et al.^[21]^ | 2022 | | 22 | | Prospective cohort | | IV | | Actifit/CMI | | 61 | |
| Spencer et al.^[22]^ | 2012 | | 12 | | case series | | IV | | Actifit/CMI | | 64 | |
| Abat et al.^[23]^ | 2012 | | 33 | | prospective cohort | | II | | allograft | | 56 | |
| Alentorn et al. | 2010 | | 15 | | retrospective cohort | | IV | | allograft | | 42 | |
| Baldairon et al.^[24]^ | 2019 | | 5 | | case series | | IV | | allograft | | 64 | |
| Cameron et al.^[25]^ | 1997 | | 67 | | retrospective cohort | | IV | | allograft | | 63 | |
| Chalmerss et al. | 2013 | | 13 | | retrospective cohort | | IV | | allograft | | 49 | |
| Chang et al.^[26]^ | 2008 | | 12 | | case series | | III | | allograft | | 64 | |
| Cvetanovich et al.^[27]^ | 2020 | | 87 | | case series | | IV | | allograft | | 42 | |
| Cole et al. | 2006 | | 40 | | prospective cohort | | IV | | allograft | | 65 | |
| Farr et al.^[28]^ | 2007 | | 36 | | prospective cohort | | IV | | allograft | | 61 | |
| Fukushima et al. | 2004 | | 40 | | case series | | IV | | allograft | | 53 | |
| Ha et al.^[29]^ | | 2011 | | 22 | | case series | | IV | | allograft | | 53 |
| Hommen et al. | | 2007 | | 22 | | retrospective cohort | | IV | | allograft | | 54 |
| Jang et al.^[30]^ | | 2015 | | 13 | | retrospective cohort | | IV | | allograft | | 65 |
| Kazi et al. | | 2015 | | 86 | | retrospective cohort | | IV | | allograft | | 49 |
| Kempshall et al.^[31]^ | | 2015 | | 60 | | prospective cohort | | III | | allograft | | 49 |
| Kim et al.^[32]^ | | 2018 | | 30 | | case series | | IV | | allograft | | 59 |
| Kim et al.^[33]^ | | 2020 | | 249 | | retrospective cohort | | III | | allograft | | 49 |
| Kocher et al. | | 2016 | | 7 | | case series | | IV | | allograft | | 56 |
| Koh et al.^[34]^ | | 2018 | | 37 | | retrospective cohort | | IV | | allograft | | 46 |
| LaPrade et al.^[35]^ | | 2010 | | 34 | | prospective cohort | | IV | | allograft | | 63 |
| Lee et al.^[36]^ | | 2017 | | 87 | | retrospective cohort | | IV | | allograft | | 64 |
| Liu et al.^[37]^ | | 2020 | | 22 | | case series | | IV | | allograft | | 65 |
| Marcacci et al. | | 2012 | | 32 | | prospective cohort | | IV | | allograft | | 61 |
| Marcacci et al. | | 2014 | | 12 | | case series | | IV | | allograft | | 67 |
| McCormick et al. | | 2014 | | 172 | | retrospective cohort | | IV | | allograft | | 67 |
| Milachowski et al.^[38]^ | | 1989 | | 22 | | case series | | IV | | allograft | | 59 |
| Noyes et al. | | 2016 | | 72 | | prospective cohort | | IV | | allograft | | 49 |
| Noyes et al.^[39]^ | | 2005 | | 38 | | case series | | IV | | allograft | | 66 |
| Parkinson et al. | | 2016 | | 71 | | prospective cohort | | III | | allograft | | 49 |
| Potter et al.^[40]^ | | 1996 | | 29 | | case series | | IV | | allograft | | 59 |
| Puzzitiello et al.^[41]^ | | 2020 | | 17 | | case series | | IV | | allograft | | 49 |
| Rath et al.^[42]^ | | 2001 | | 27 | | prospective cohort | | IV | | allograft | | 59 |
| Roumazeille et al.^[43]^ | | 2015 | | 22 | | retrospective cohort | | IV | | allograft | | 48 |
| Rue et al.^[44]^ | | 2008 | | 31 | | case series | | IV | | allograft | | 59 |
| Ryu et al.^[45]^ | | 2002 | | 26 | | retrospective cohort | | IV | | allograft | | 49 |
| Saltzman et al. | | 2017 | | 40 | | prospective cohort | | IV | | allograft | | 41 |
| Searle et al^[46]^ | | 2020 | | 43 | | case series | | IV | | allograft | | 59 |
| Sekiya et al.^[47]^ | | 2003 | | 28 | | retrospective cohort | | IV | | allograft | | 59 |
| Stone et al.^[48]^ | | 2015 | | 49 | | prospective cohort | | IV | | allograft | | 58 |
| van Arkel et al. | | 2002 | | 63 | | prospective cohort | | IV | | allograft | | 65 |
| Van Der Straeten  et al. | | 2016 | | 329 | | retrospective cohort | | IV | | allograft | | 64 |
| van der wal et al.^[49]^ | | 2020 | | 111 | | prospective cohort | | III | | allograft | | 45 |
| verdonk et al. | | 2005 | | 61 | | prospective cohort | | IV | | allograft | | 52 |
| Vundelinckx et al. | | 2014 | | 30 | | retrospective cohort | | IV | | allograft | | 63 |
| Waterman et al. | | 2016 | | 230 | | retrospective cohort | | IV | | allograft | | 41 |
| Wirth et al. et al. | | 2002 | | 23 | | prospective cohort | | IV | | allograft | | 61 |
| Yoldas et al.^[50]^ | | 2003 | | 12 | | retrospective cohort | | IV | | allograft | | 51 |
| Yoon et al.^[51]^ | | 2014 | | 30 | | retrospective cohort | | IV | | allograft | | 68 |
| Yoon et al. | | 2014 | | 56 | | retrospective cohort | | III | | allograft | | 69 |
| Zaffagnini et al.^[52]^ | | 2016 | | 147 | | retrospective cohort | | IV | | allograft | | 56 |
| Zhang et al.^[53]^ | | 2012 | | 19 | | prospective cohort | | IV | | allograft | | 52 |
| CMS: Coleman Methodological Score. | | | | | |  | | | |  | |  |

[1] Bouyarmane H, Beaufils P, Pujol N, et al. Polyurethane scaffold in lateral meniscus segmental defects: clinical outcomes at 24 months follow-up. *Orthop Traumatol Surg Res*. Feb 2014;100(1):153-157. doi:10.1016/j.otsr.2013.10.011

[2] Condello V, Dei Giudici L, Perdisa F, et al. Polyurethane scaffold implants for partial meniscus lesions: delayed intervention leads to an inferior outcome. *Knee Surg Sports Traumatol Arthrosc*. Jan 2021;29(1):109-116. doi:10.1007/s00167-019-05760-4

[3] Efe T, Getgood A, Schofer MD, et al. The safety and short-term efficacy of a novel polyurethane meniscal scaffold for the treatment of segmental medial meniscus deficiency. *Knee Surg Sports Traumatol Arthrosc*. Sep 2012;20(9):1822-1830. doi:10.1007/s00167-011-1779-3

[4] Faivre B, Bouyarmane H, Lonjon G, et al. Actifit® scaffold implantation: Influence of preoperative meniscal extrusion on morphological and clinical outcomes. *Orthop Traumatol Surg Res*. Oct 2015;101(6):703-708. doi:10.1016/j.otsr.2015.06.016

[5] Filardo G, Kon E, Perdisa F, et al. Polyurethane-based cell-free scaffold for the treatment of painful partial meniscus loss. *Knee Surg Sports Traumatol Arthrosc*. Feb 2017;25(2):459-467. doi:10.1007/s00167-016-4219-6

[6] Gelber PE, Torres-Claramunt R, Poggioli F, et al. Polyurethane Meniscal Scaffold: Does Preoperative Remnant Meniscal Extrusion Have an Influence on Postoperative Extrusion and Knee Function? *J Knee Surg*. Dec 2021;34(14):1555-1559. doi:10.1055/s-0040-1710377

[7] Haspl M, Trsek D, Lovric D, et al. Functional and magnetic resonance imaging outcome after polyurethane meniscal scaffold implantation following partial meniscectomy. *Int Orthop*. Apr 2021;45(4):971-975. doi:10.1007/s00264-020-04844-y

[8] Leroy A, Beaufils P, Faivre B, et al. Actifit(®) polyurethane meniscal scaffold: MRI and functional outcomes after a minimum follow-up of 5 years. *Orthop Traumatol Surg Res*. Jun 2017;103(4):609-614. doi:10.1016/j.otsr.2017.02.012

[9] Monllau JC, Poggioli F, Erquicia J, et al. Magnetic Resonance Imaging and Functional Outcomes After a Polyurethane Meniscal Scaffold Implantation: Minimum 5-Year Follow-up. *Arthroscopy*. May 2018;34(5):1621-1627. doi:10.1016/j.arthro.2017.12.019

[10] Olivos-Meza A, Pérez Jiménez FJ, Granados-Montiel J, et al. First Clinical Application of Polyurethane Meniscal Scaffolds with Mesenchymal Stem Cells and Assessment of Cartilage Quality with T2 Mapping at 12 Months. *Cartilage*. Dec 2021;13(1_suppl):197s-207s. doi:10.1177/1947603519852415

[11] Schüttler KF, Pöttgen S, Getgood A, et al. Improvement in outcomes after implantation of a novel polyurethane meniscal scaffold for the treatment of medial meniscus deficiency. *Knee Surg Sports Traumatol Arthrosc*. Jul 2015;23(7):1929-1935. doi:10.1007/s00167-014-2977-6

[12] Verdonk P, Beaufils P, Bellemans J, et al. Successful treatment of painful irreparable partial meniscal defects with a polyurethane scaffold: two-year safety and clinical outcomes. *Am J Sports Med*. Apr 2012;40(4):844-853. doi:10.1177/0363546511433032

[13] Hirschmann MT, Keller L, Hirschmann A, et al. One-year clinical and MR imaging outcome after partial meniscal replacement in stabilized knees using a collagen meniscus implant. *Knee Surg Sports Traumatol Arthrosc*. Mar 2013;21(3):740-747. doi:10.1007/s00167-012-2259-0

[14] Kovacs BK, Huegli R, Harder D, et al. MR variability of collagen meniscal implant remodelling in patients with good clinical outcome. *Knee Surg Sports Traumatol Arthrosc*. Jan 2021;29(1):90-99. doi:10.1007/s00167-019-05715-9

[15] Linke RD, Ulmer M, Imhoff AB. Replacement of the meniscus with a collagen implant (CMI). *Oper Orthop Traumatol*. Dec 2006;18(5-6):453-462. doi:10.1007/s00064-006-1188-9

[16] Monllau JC, Gelber PE, Abat F, et al. Outcome after partial medial meniscus substitution with the collagen meniscal implant at a minimum of 10 years' follow-up. *Arthroscopy*. Jul 2011;27(7):933-943. doi:10.1016/j.arthro.2011.02.018

[17] Rodkey WG, Steadman JR, Li ST. A clinical study of collagen meniscus implants to restore the injured meniscus. *Clin Orthop Relat Res*. Oct 1999;(367 Suppl):S281-292. doi:10.1097/00003086-199910001-00027

[18] Schenk L, Bethge L, Hirschmann A, et al. Ongoing MRI remodeling 3-7 years after collagen meniscus implantation in stable knees. *Knee Surg Sports Traumatol Arthrosc*. Apr 2020;28(4):1099-1104. doi:10.1007/s00167-019-05714-w

[19] Stone KR, Steadman JR, Rodkey WG, et al. Regeneration of meniscal cartilage with use of a collagen scaffold. Analysis of preliminary data. *J Bone Joint Surg Am*. Dec 1997;79(12):1770-1777. doi:10.2106/00004623-199712000-00002

[20] Zaffagnini S, Marcheggiani Muccioli GM, Bulgheroni P, et al. Arthroscopic collagen meniscus implantation for partial lateral meniscal defects: a 2-year minimum follow-up study. *Am J Sports Med*. Oct 2012;40(10):2281-2288. doi:10.1177/0363546512456835

[21] Reale D, Lucidi GA, Grassi A, et al. A Comparison Between Polyurethane and Collagen Meniscal Scaffold for Partial Meniscal Defects: Similar Positive Clinical Results at a Mean of 10 Years of Follow-Up. *Arthroscopy*. Apr 2022;38(4):1279-1287. doi:10.1016/j.arthro.2021.09.011

[22] Spencer SJ, Saithna A, Carmont MR, et al. Meniscal scaffolds: early experience and review of the literature. *Knee*. Dec 2012;19(6):760-765. doi:10.1016/j.knee.2012.01.006

[23] Abat F, Gelber PE, Erquicia JI, et al. Suture-only fixation technique leads to a higher degree of extrusion than bony fixation in meniscal allograft transplantation. *Am J Sports Med*. Jul 2012;40(7):1591-1596. doi:10.1177/0363546512446674

[24] Baldairon F, Toanen C, Pujol N. Functional and anatomical outcomes of single-stage arthroscopic bimeniscal replacement. *Orthop Traumatol Surg Res*. Nov 2019;105(7):1383-1387. doi:10.1016/j.otsr.2019.08.010

[25] Cameron JC, Saha S. Meniscal allograft transplantation for unicompartmental arthritis of the knee. *Clin Orthop Relat Res*. Apr 1997;(337):164-171. doi:10.1097/00003086-199704000-00018

[26] Chang HC, Teh KL, Leong KL, et al. Clinical evaluation of arthroscopic-assisted allograft meniscal transplantation. *Ann Acad Med Singap*. Apr 2008;37(4):266-272.

[27] Cvetanovich GL, Christian DR, Garcia GH, et al. Return to Sport and Patient Satisfaction After Meniscal Allograft Transplantation. *Arthroscopy*. Sep 2020;36(9):2456-2463. doi:10.1016/j.arthro.2020.04.034

[28] Farr J, Rawal A, Marberry KM. Concomitant meniscal allograft transplantation and autologous chondrocyte implantation: minimum 2-year follow-up. *Am J Sports Med*. Sep 2007;35(9):1459-1466. doi:10.1177/0363546507301257

[29] Ha JK, Sung JH, Shim JC, et al. Medial meniscus allograft transplantation using a modified bone plug technique: clinical, radiologic, and arthroscopic results. *Arthroscopy*. Jul 2011;27(7):944-950. doi:10.1016/j.arthro.2011.02.013

[30] Jang KM, Wang JH. Lateral meniscus allograft transplantation using a single-incision technique. *Knee Surg Sports Traumatol Arthrosc*. Jan 2015;23(1):258-263. doi:10.1007/s00167-013-2464-5

[31] Kempshall PJ, Parkinson B, Thomas M, et al. Outcome of meniscal allograft transplantation related to articular cartilage status: advanced chondral damage should not be a contraindication. *Knee Surg Sports Traumatol Arthrosc*. Jan 2015;23(1):280-289. doi:10.1007/s00167-014-3431-5

[32] Kim JH, Lee S, Ha DH, et al. The effects of graft shrinkage and extrusion on early clinical outcomes after meniscal allograft transplantation. *J Orthop Surg Res*. Jul 20 2018;13(1):181. doi:10.1186/s13018-018-0892-0

[33] Kim C, Bin SI, Kim JM, et al. Medial and Lateral Meniscus Allograft Transplantation Showed No Difference With Respect to Graft Survivorship and Clinical Outcomes: A Comparative Analysis With a Minimum 2-Year Follow-Up. *Arthroscopy*. Dec 2020;36(12):3061-3068. doi:10.1016/j.arthro.2020.07.025

[34] Koh YG, Kim YS, Kwon OR, et al. Comparative Matched-Pair Analysis of Keyhole Bone-Plug Technique Versus Arthroscopic-Assisted Pullout Suture Technique for Lateral Meniscal Allograft Transplantation. *Arthroscopy*. Jun 2018;34(6):1940-1947. doi:10.1016/j.arthro.2018.01.053

[35] LaPrade RF, Wills NJ, Spiridonov SI, et al. A prospective outcomes study of meniscal allograft transplantation. *Am J Sports Med*. Sep 2010;38(9):1804-1812. doi:10.1177/0363546510368133

[36] Lee BS, Bin SI, Kim JM, et al. Survivorship After Meniscal Allograft Transplantation According to Articular Cartilage Status. *Am J Sports Med*. Apr 2017;45(5):1095-1101. doi:10.1177/0363546516682235

[37] Liu JN, Agarwalla A, Garcia GH, et al. Return to Sport and Work After High Tibial Osteotomy With Concomitant Medial Meniscal Allograft Transplant. *Arthroscopy*. Nov 2019;35(11):3090-3096. doi:10.1016/j.arthro.2019.05.053

[38] Milachowski KA, Weismeier K, Wirth CJ. Homologous meniscus transplantation. Experimental and clinical results. *Int Orthop*. 1989;13(1):1-11. doi:10.1007/bf00266715

[39] Noyes FR, Barber-Westin SD, Rankin M. Meniscal transplantation in symptomatic patients less than fifty years old. *J Bone Joint Surg Am*. Sep 2005;87 Suppl 1(Pt 2):149-165. doi:10.2106/jbjs.E.00347

[40] Potter HG, Rodeo SA, Wickiewicz TL, et al. MR imaging of meniscal allografts: correlation with clinical and arthroscopic outcomes. *Radiology*. Feb 1996;198(2):509-514. doi:10.1148/radiology.198.2.8596858

[41] Puzzitiello RN, Liu JN, Garcia GH, et al. Return to Sport and Outcomes After Concomitant Lateral Meniscal Allograft Transplant and Distal Femoral Varus Osteotomy. *Arthroscopy*. Jan 2020;36(1):253-260. doi:10.1016/j.arthro.2019.07.022

[42] Rath E, Richmond JC, Yassir W, et al. Meniscal allograft transplantation. Two- to eight-year results. *Am J Sports Med*. Jul-Aug 2001;29(4):410-414. doi:10.1177/03635465010290040401

[43] Roumazeille T, Klouche S, Rousselin B, et al. Arthroscopic meniscal allograft transplantation with two tibia tunnels without bone plugs: evaluation of healing on MR arthrography and functional outcomes. *Knee Surg Sports Traumatol Arthrosc*. Jan 2015;23(1):264-269. doi:10.1007/s00167-013-2476-1

[44] Rue JP, Yanke AB, Busam ML, et al. Prospective evaluation of concurrent meniscus transplantation and articular cartilage repair: minimum 2-year follow-up. *Am J Sports Med*. Sep 2008;36(9):1770-1778. doi:10.1177/0363546508317122

[45] Ryu RK, Dunbar VW, Morse GG. Meniscal allograft replacement: a 1-year to 6-year experience. *Arthroscopy*. Nov-Dec 2002;18(9):989-994. doi:10.1053/jars.2002.36104

[46] Searle H, Asopa V, Coleman S, et al. The results of meniscal allograft transplantation surgery: what is success? *BMC Musculoskelet Disord*. Mar 12 2020;21(1):159. doi:10.1186/s12891-020-3165-0

[47] Sekiya JK, Giffin JR, Irrgang JJ, et al. Clinical outcomes after combined meniscal allograft transplantation and anterior cruciate ligament reconstruction. *Am J Sports Med*. Nov-Dec 2003;31(6):896-906. doi:10.1177/03635465030310062701

[48] Stone KR, Pelsis JR, Surrette ST, et al. Meniscus transplantation in an active population with moderate to severe cartilage damage. *Knee Surg Sports Traumatol Arthrosc*. Jan 2015;23(1):251-257. doi:10.1007/s00167-014-3246-4

[49] van der Wal RJP, Nieuwenhuijse MJ, Spek RWA, et al. Meniscal allograft transplantation in The Netherlands: long-term survival, patient-reported outcomes, and their association with preoperative complaints and interventions. *Knee Surg Sports Traumatol Arthrosc*. Nov 2020;28(11):3551-3560. doi:10.1007/s00167-020-06276-y

[50] Yoldas EA, Sekiya JK, Irrgang JJ, et al. Arthroscopically assisted meniscal allograft transplantation with and without combined anterior cruciate ligament reconstruction. *Knee Surg Sports Traumatol Arthrosc*. May 2003;11(3):173-182. doi:10.1007/s00167-003-0362-y

[51] Yoon KH, Lee SH, Park SY, et al. Meniscus allograft transplantation: a comparison of medial and lateral procedures. *Am J Sports Med*. Jan 2014;42(1):200-207. doi:10.1177/0363546513509057

[52] Zaffagnini S, Grassi A, Marcheggiani Muccioli GM, et al. Is Sport Activity Possible After Arthroscopic Meniscal Allograft Transplantation? Midterm Results in Active Patients. *Am J Sports Med*. Mar 2016;44(3):625-632. doi:10.1177/0363546515621763

[53] Zhang H, Liu X, Wei Y, et al. Meniscal allograft transplantation in isolated and combined surgery. *Knee Surg Sports Traumatol Arthrosc*. Feb 2012;20(2):281-289. doi:10.1007/s00167-011-1572-3
